# Food Insecurity and Dental Caries in U.S. and South Korean Older Adults Ages 60+

**DOI:** 10.1093/geroni/igaa057.774

**Published:** 2020-12-16

**Authors:** Donald Chi, Yujin Kim, JoAnna Scott

**Affiliations:** 1 University of Washington, Seattle, Washington, United States; 2 Kangwon National University, Chuncheon, Kangwon-do, Republic of Korea; 3 UMKC, Kansas City, Missouri, United States

## Abstract

There are no studies on the association between food insecurity and dental caries (tooth decay) in older adults. We analyzed nationally representative data from two countries – the U.S. and South Korea – to test the hypothesis that food insecurity would be positively associated with tooth decay in older adults ages 60+. The U.S. analyses focused on National Health and Nutrition Examination Survey data from three cycles (2011-12, 2013-14, and 2015-16) and the South Korean analyses focused on Korean National Health and Nutrition Examination Survey data from one cycle (2013-2015). Food insecurity was defined using the 18-item USDA Household Food Security Survey and a validated Korean version of the same survey. Tooth decay was defined as having at least one decayed tooth (no/yes). We ran logistic regression models to generate odds ratios and 95% confidence intervals (CI). The percentage of fully food secure older adults was 74.0% in the U.S. and 82.2% in South Korea. Twenty-three percent of U.S. older adults and 28% of South Korean older adults had at least one decayed tooth. Older adults in the U.S. with food insecurity were 2.03 times as likely to have untreated tooth decay (95% CI: 1.43, 2.87; P<0.0001). Findings were similar for South Korean older adults. Food insecurity is significantly associated with untreated tooth decay in both U.S. and South Korean older adults. Future research should identify the aspects of food insecurity that increase older adults’ risk for tooth decay, with an emphasis on those that are amenable to population-based interventions.

